# Plasma albumin determination on the Cobas Bio centrifugal analyser using bromocresol green

**DOI:** 10.1155/S1463924683000371

**Published:** 1983

**Authors:** J. R. Cowie, G. O. Evans

**Affiliations:** 1 Department of Toxicology Wellcome Research Laboratories Langley Court Beckenham, Kent Dartford UK; 2 Wellcome's laboratories in Dartford Kent UK


Journal of Automatic Chemistry, Volume 5, Number 3 (July–September 1983), pages 153–154

### Plasma albumin determination on the Cobas Bio centrifugal analyser using bromocresol green

J. R. Cowie and G. O. Evans

*Department of Toxicology, Wellcome Research Laboratories, Langley Court, Beckenham, Kent, UK**

### Introduction

The bromocresol green (BCG) method was introduced by Rodkey [1] in 1965 for determination of serum and plasma albumin and it is still widely used—despite criticism of the available procedures [2, 3, 4 and 5]. In 1976, Savory *et al.* [6] adopted a method for use in centrifugal analysis demonstrating good precision and accuracy. Although bromocresol purple has been suggested as an alternative dye [7 and 8], this dye is not suitable for samples from some animal species nor for quality-control sera (for example bovine sera). The lack of readily available specific antisera for some animal species, and the unsuitability of bromocresol purple, led the authors to develop an automated BCG method using the Cobas Bio (Roche) centrifugal analyser [9] and to compare this method with an immunoturbidimetric method for human albumin using the same instrument. Centrifugal analysers permit very rapid absorbance measurements to be made, thus avoiding the slower reactions resulting from the binding of certain alpha- and betaglobulins with BCG. The over-estimation of plasma serum albumin at lower concentrations may be avoided by adjusting the assay conditions.

### Materials and methods

### *BCG method*

The reagents used were as follows:

(1) Citrate buffer solution, pH 3·8, obtained from BDH Chemicals Ltd, Poole, UK—product number 22115.
(2) Bromocrescol green, obtained from BDH Chemicals Ltd. A solution containing 2 mg/ml BCG in the citrate buffer solution is prepared and stored at ambient temperature. This reagent has been found to be stable for at least three months.
(3) Wellcomtrol Survey Validated Reference Serum from Wellcome Diagnostics, Dartford, Kent, UK.
(4) Preciset Protein Standards (bovine albumin), obtained from BCL, Lewes, East Sussex, UK.

The instrument settings finally chosen for the BCG method using the Cobas Bio centrifugal analyser are presented in table 1; this analysis programme allows for rapid absorbance measurements to be made following the addition of BCG reagent which acts as a 'start reagent' when added to the diluted samples.

### *Immunoturbidimetric method*

Reagents used were:

(1) 0·9% isotonic saline.
(2) Polyethylene glycol (PEG) Diluent 6000, obtained from BCL, is diluted by adding 5 ml to 95 ml isotonic saline before use.
(3) Anti-human albumin serum solution from BCL is diluted 1 + 60 with the diluted PEG reagent.
(4) Human albumin obtained from Calbiochem—Behring Corporation.

The immunoturbidimetric method [10] requires that 5 μl of diluted serum or plasma (one volume of serum or plasma plus 200 volumes of PEG working solution) be added to 255 μl of diluted anti-serum at 25°C. The concentration values for albumin are determined from changes in absorbance at 340 nm, monitored at 10 s intervals for a period of 200 s. The frequent monitoring of the reaction allows for the detection of antigen excess reactions, and variability between different batches of antisera and polyethylene glycol reagents. When $N = 18$ samples, within-batch coefficients of variation of 3·82% at 30 g/l and 3·47% at 50 g/l were obtained.

*Table 1. Cobas Bio settings used for the determination of plasma albumin using bromocresol green.*

| | |
|---|---|
| (1) Units | g/l |
| (2) Calculation factor | 0 |
| (3) Standard 1 concentration | |
| (4) Standard 2 concentration | Assigned |
| (5) Standard 3 concentration | value |
| (6) Limit | 60 |
| (7) Temperature (°C) | 30·0 |
| (8) Type of analysis | 6 |
| (9) Wavelength (nm) | 620 |
| (10) Sample volume (μl) | 05 |
| (11) Diluent volume (μl) | 30 |
| (12) Reagent volume (μl) | 200 |
| (13) Incubation time (s) | 10 |
| (14) Start reagent volume (μl) | 20 |
| (15) Time of first reading (s) | 10·0 |
| (16) Time interval (s) | 10 |
| (17) Number of readings | 01 |
| (18) Blanking mode | 1 |
| (19) Print-out mode | 1 |

### Results

Using Preciset protein and human albumin standards, ranging between 10 and 70 g/l in ascending and descending order of concentration, the BCG assay was found to be linear between 20 and 60 g/l. Alterations of the dye concentrations caused marked shifts in the relationship between absorbance and concentration, resulting in either over- or under-estimation of albumin concentration levels. Using the procedure of Broughton *et al.* [11],

* *J. R. Cowie is now based at Wellcome's laboratories in Dartford, Kent, UK.*

carry-over between samples was not evident when 20 g/l and 70 g/l standards were used to take readings, with interaction $(K) = -0{\cdot}0031$. When $N=25$ samples, within-batch CVs of 1·36% at 27 g/l and 1·05% at 40·7 g/l were obtained. Between-batch reproducibility was found to be 2·45% at 27 g/l and 2·41% at 40·7 g/l using Wellcomtrol Survey Validated Reference Serum ($N=40$).

The accuracy of the BCG method was assessed by a comparison of the method values with values obtained using the immunoturbidimetric procedure for human plasma samples, and also with the consensus mean values found in the National External Quality Assurance Scheme (NEQAS) and the Wellcome Group Quality Control Programme (WGQCP). Results obtained by BCG and immunoturbidimetric procedures showed good agreement over the range 22–57 g/l for 30 human samples (see figure 1). For eight NEQAS samples distributed in 1982 and analysed on the day of reconstitution, all results were within 1 standard deviation of the recalculated overall mean values, with six of the results within 0·5 SD.

For 12 WGQCP samples the bias observed was 0·119, and a precision value of 1·18 was obtained, compared with an overall precision value of 1·59 for 993 laboratories. For 16 other WGQCP samples, close agreement was also achieved where the correlation coefficient was 0·99 ($y=1{\cdot}04x-1{\cdot}65$).

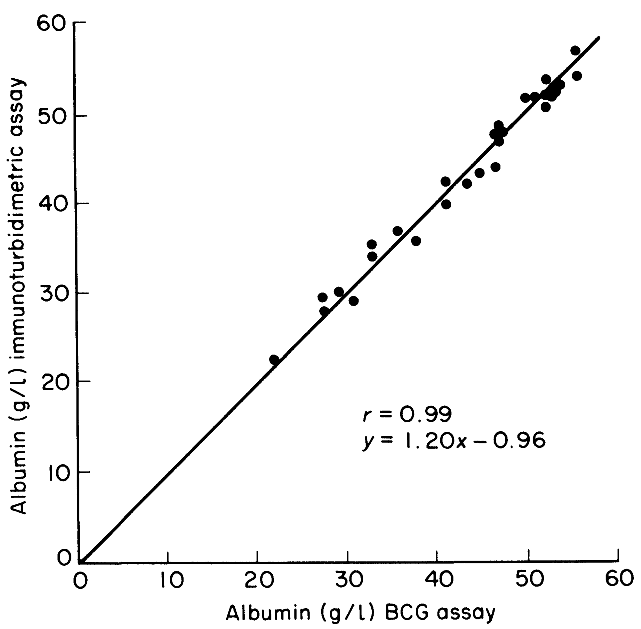

*Figure 1. Comparison between BCG and immunoturbidimetric values for human samples.*

### Discussion

An albumin method using bromocresol green and a Cobas Bio centrifugal analyser has been developed for use with plasma samples from several different animal species, over the range 25 to 60 g/l. The procedure shows good agreement with values obtained by an immunoturbidimetric method. Performance of the BCG method in assaying samples from external quality assessment schemes was found to be acceptable. The precision of the assay was slightly better than with the immunoturbidimetric procedure used for this comparison, and the determined coefficients of variation were similar to those obtained by Izquierdo *et al.* [12] who used a Cobas Bio centrifugal analyser and a succinate, rather than a citrate buffer diluent for albumin determinations. The recent advances in laboratory analysers, particularly centrifugal analysers, have led to improvements of albumin determinations using bromocresol green; it is now possible to make rapid absorbance measurements following the addition of dye to sample, thus reducing the effect of reactions between the dye and other protein fractions.

### References

1. Rodkey, F. L., *Clinical Chemistry*, **11** (1965), 478.
2. Gustafsson, J. E. C., *Clinical Chemistry*, **22** (1976), 616.
3. Webster, D., Bignell, A. H. C. and Attwood, E. C., *Clinica Chimica Acta*, **53** (1974), 101.
4. Duggan, J. and Duggan, P. F., *Clinical Chemistry*, **28** (1982), 1407.
5. Peters, T. Jr., *Clinical Chemistry*, **23** (1977), 5.
6. Savory, J., Heintges, M. G., Sonowane, M. and Cross, R. E., *Clinical Chemistry*, **22** (1976), 1102.
7. Louderback, A., Mealey, E. H. and Taylor, N. A., *Clinical Chemistry*, **14** (1968), 793.
8. Pinnell, A. E. and Northam, B. E., *Clinical Chemistry*, **24** (1978), 80.
9. Bais, R., White, R. G., Elston, D. and Geary, T. D., *Journal of Automatic Chemistry*, **4** (1982), 21.
10. Cowie, J. R., Unpublished data.
11. Broughton, P. M. G., Buttolph, M. A., Gowenlock, A. M., Neill, D. W. and Skentelbery, R. G., *Journal of Clinical Pathology*, **22** (1969), 278.
12. Izquierdo, J. M., Sotorrio, P., Alvarez-Uría, J. and Romeo, D., *Clinical Chemistry*, **28** (1982), 1629.

### FORTHCOMING PAPERS

Among those articles to be published in *Journal of Automatic Chemistry*, Vol. 5, No. 4 (October–December 1983) are:

‘Six years’ experience of in-house maintenance on clinical chemistry autoanalysers’ by D. J. Wyper *et al.* (The authors show that the in-house scheme run by the West of Scotland Health Boards has been of considerable financial benefit, also that it has improved technical expertise within the NHS. The scheme is fully costed to allow comparison with commercial alternatives.)

‘Automated determination of zinc and copper in plasma’ by B. Sampson. (Estimation of copper and zinc concentrations in plasma and serum is becoming a commonly requested analysis. The continuous-flow method described is readily adaptable to most clinical laboratories.)

